# Supplementary material for: Architectural principles for Hfq/Crc-mediated regulation of gene expression
Source: eLife. 2019 Feb 13;8:e43158. doi: 10.7554/eLife.43158 (PMC6422490; doi:10.7554/eLife.43158)
Supplement: Supplementary file 1. — (A) Summary of the data collection parameters and the reconstruction and refinement statistics for the Hfq:Crc:RNA complexes. (B) List of the plasmids and strains used in this study. (C) Oligonucleotides used in this study. [file elife-43158-supp1.docx]

**Supplementary file 1A.** Cryo-EM data collection and refinement statistics for Hfq/Crc/RNA structures

BS^3^ crosslinked Not crosslinked

PDB codes 6FUS,6FYN,6FXZ

EMDB codes 4320, 4326, 4325

**Data collection**

EM equipment Titan Krios G2, FEI Titan Krios G2, FEI

Voltage (kV) 300 300

Detector Falcon III Falcon III

Pixel size (Å) 1.09 1.09

Electron dose (e-/Å^2^/fraction) 0.37 0.40

Defocus range, step (µm) -1.25 to -3, δ=0.25 -1.25 to -3, δ=0.25

**Reconstruction**

Software RELION v 3b RELION v2.1

Complex (Hfq:Crc:RNA) 2:2:2 2:3:2 2:4:2 2:2:2 2:3:2 2:4:2

Molecular mass (kDa) 192 222 252 192 222 252

Number of particles used 73,187 45,311 35,376 25,408 18,898 14,900

Angular accuracies (°) 0.72 0.75 0.66 1.65 1.71 1.58

Offsets (pixels) 0.30 0.32 0.30 0.652 0.681 0.657

Symmetry C2 C1 C2 C2 C1 C2

Final resolution (Å) 3.1 3.4 3.2 4.4 4.5 4.4

Map-sharpening B factor (Å^2^) -97 -100 -82 -189 -172 -173

Non-hydrogen atoms 11466 13647 15770 11466 13647 15770

Protein residues 1292 1458 1820 1292 1558 1820

RNA bases 18 18 18 18 18 18

**Refinement**

Software PhenixRSRef

Model-to-Map Correlation Coef. 0.79 0.77 0.76

**Model Validation**

MolProbity score 1.64 1.77 1.69

EMRinger 3.8 3.4 3.2

All-atom clash score 5.07 5.44 5.26

***Ramachandran statistics*** (%)

Favored (overall) 94.57 92.64 93.81

Allowed (overall) 5.27 7.30 6.19

Outlier (overall) 0.16 0.06 0.0

***RMS deviations***

Bond length (Å ) 0.004 0.007 0.005

Bond angle (°) 0.84 0.88 0.908

**Validation (RNA)**

Correct sugar puckers (%) 89 89 89

Good backbone conformation (%) 39 39 39

**Supplementary file 1B.** Strains and plasmids used in this study

| **Strain/plasmid** | **Genotype/relevant features** | **Source/reference** |
| --- | --- | --- |
| ***P. aeruginosa*** |  |  |
| PAO1 |  | (Holloway et al., 1979) |
| PAO1Δ*crc* | PAO6673, in frame deletion of *crc* deletion | (Sonnleitner and Bläsi, 2014) |
| ***E. coli*** |  |  |
| XL1-Blue | *recA1 endA1 gyrA96 thi-1 hsdR17*(rK-, mK+) *supE44 relA1 lac* [F' *proAB* *lacI^q^ lacZ*ΔM15::Tn*10*(Tc^r^)] | Stratagene |
| BL21(DE3) | *F^-^, ompT, hsdS_B_(r_B_-, m_B_-), dcm, gal, λ*(DE3) | Novagen |
| **Plasmids** |  |  |
| pETM14lic-His_6_Crc | Encodes Crc with a N-terminal cleavable His_6_-tag. Transcription of the *crc* gene is driven by a T7 promoter. Kan^R^ | (Milojevic et al., 2013) |
| pME9655 | Encoding the translational *amiE::lacZ* reporter gene. Tc^r^ | (Sonnleitner and Bläsi, 2014) |
| pME4510*crc*_Flag_ | pME4510 carrying PAO1 *crc* fused to a Flag-tag encoding sequence under control of its authentic promoter | (Sonnleitner et al., 2018) |
| pME4510*crc*_(R140E)Flag_ | pME4510*crc*_Flag_ derivative encoding the Crc_(R140E)_ variant | This study |
| pME4510*crc*_(E142R)Flag_ | pME4510*crc*_Flag_ derivative encoding the Crc_(E142R)_ variant | This study |
| pME4510*crc*_(R229E)Flag_ | pME4510*crc*_Flag_ derivative encoding the Crc_(R229E)_ variant | This study |
| pME4510*crc*_(R230E)Flag_ | pME4510*crc*_Flag_ derivative encoding the Crc_(R230E)_ variant | This study |
| pME4510*crc*_(E142R, R229E)Flag_ | pME4510*crc*_Flag_ derivative encoding the Crc_(E142R, R229E)_ variant | This study |
| pME4510*crc*_(E142R, R230E)Flag_ | pME4510*crc*_Flag_ derivative encoding the Crc_(E142R, R230E)_ variant | This study |
| pME4510*crc*_(E142R, R229E, R230E)Flag_ | pME4510*crc*_Flag_ derivative encoding the Crc(_E142R, R229E, R230E)_ variant | This study |
| pME4510*crc*_(E142A, R229E, R230E)Flag_ | pME4510*crc*_Flag_ derivative encoding the Crc(_E142A, R229E, R230E)_ variant | This study |
| pME4510*crc*_(E193R)Flag_ | pME4510*crc*_Flag_ derivative encoding the Crc_(E193R)_ variant | This study |
| pME4510*crc*_(E193R, R230E)Flag_ | pME4510*crc*_Flag_ derivative encoding the Crc_(E193R, R230E)_ variant | This study |
| pME4510*crc*_(E193A, R230E)Flag_ | pME4510*crc*_Flag_ derivative encoding the Crc_(E193A, R230E)_ variant | This study |
| pETM14lic-His_6_Crc_R140E_ | pETM14lic-His_6_Crc derivative encoding the Crc_(R140E)_ variant | This study |
| pETM14lic-His_6_Crc_E142R_ | pETM14lic-His_6_Crc derivative encoding the Crc_E142R)_ variant | This study |
| pETM14lic-His_6_Crc_R230E_ | pETM14lic-His_6_Crc derivative encoding the Crc_(R230E)_ variant | This study |

**Supplementary file 1C. Oligonucleotides used in this study**

| **Name** | **Sequence^a^** | **Mutation/orientation** |
| --- | --- | --- |
| N142 | GCGTCGCAAGgaaCGCGAATACATC | Crc_R140E_/forward |
| O142 | GATGTATTCGCgttCCTTGCGACGC | Crc_R140E_/reverse |
| P142 | CAAGCGCCGCcgcTACATCTACTGC | Crc_E142R_/forward |
| Q142 | GCAGTAGATGTAgcgGCGGCGCTTG | Crc_E142R_/reverse |
| L142 | CGCCCTGCGCcgcGTCAGCCGC | Crc_E193R_/forward |
| M142 | GCGGCTGACgcgGCGCAGGGCG | Crc_E193R_/reverse |
| A145 | CCCCGGCCTAgaaCGCTTCGTGCGC | Crc_R229E_/forward |
| B145 | GCGCACGAAGCGttcTAGGCCGGGG | Crc_R229E_/reverse |
| R142 | CGGCCTACGCgaaTTCGTGCGCAAC | Crc_R230E_/forward |
| S142 | GTTGCGCACGAAttcGCGTAGGCCG | Crc_R230E_/reverse |
| I146 | CCCCGGCCTAgaagaaTTCGTGCGCAAC | Crc_R299E,R230E_/forward |
| J146 | GTTGCGCACGAAttcttcTAGGCCGGGG | Crc_R299E,R230E_/reverse |
| U173 | CAAGCGCCGCGccTACATCTACTGC | Crc_E142A_/forward |
| V173 | GCAGTAGATGTAggCGCGGCGCTTG | Crc_E142A_/reverse |
| W173 | CGCCCTGCGCGccGTCAGCCGC | Crc_E193A_/forward |
| X173 | GCGGCTGACggCGCGCAGGGCG | Crc_E193A_/reverse |

^a^ mutated sequences are shown in small letters
